# Assessing the role of surface layer and molecular probe size in diffusion within meniscus tissue

**DOI:** 10.1371/journal.pone.0301432

**Published:** 2024-04-16

**Authors:** Gabi Schwartz, Thomas M. Best, Cheng-Bang Chen, Francesco Travascio, Alicia R. Jackson

**Affiliations:** 1 Department of Biomedical Engineering, University of Miami, Coral Gables, FL, United States of America; 2 Department of Orthopaedic Surgery, University of Miami, Miami, FL, United States of America; 3 UHealth Sports Medicine Institute, Coral Gables, FL, United States of America; 4 Department of Industrial and Systems Engineering, University of Miami, Coral Gables, FL, United States of America; 5 Department of Mechanical and Aerospace Engineering, University of Miami, Coral Gables, FL, United States of America; 6 Max Biedermann Institute for Biomechanics at Mount Sinai Medical Center, Miami Beach, FL, United States of America; Drexel University, UNITED STATES

## Abstract

Diffusion within extracellular matrix is essential to deliver nutrients and larger metabolites to the avascular region of the meniscus. It is well known that both structure and composition of the meniscus vary across its regions; therefore, it is crucial to fully understand how the heterogenous meniscal architecture affects its diffusive properties. The objective of this study was to investigate the effect of meniscal region (core tissue, femoral, and tibial surface layers) and molecular weight on the diffusivity of several molecules in porcine meniscus. Tissue samples were harvested from the central area of porcine lateral menisci. Diffusivity of fluorescein (MW 332 Da) and three fluorescence-labeled dextrans (MW 3k, 40k, and 150k Da) was measured via fluorescence recovery after photobleaching. Diffusivity was affected by molecular size, decreasing as the Stokes’ radius of the solute increased. There was no significant effect of meniscal region on diffusivity for fluorescein, 3k and 40k dextrans (p>0.05). However, region did significantly affect the diffusivity of 150k Dextran, with that in the tibial surface layer being larger than in the core region (p = 0.001). Our findings contribute novel knowledge concerning the transport properties of the meniscus fibrocartilage. This data can be used to advance the understanding of tissue pathophysiology and explore effective approaches for tissue restoration.

## Introduction

The meniscus fibrocartilage plays an important role in the healthy functioning of the knee joint by maintaining efficient joint performance and protecting the neighboring femoral and tibial surfaces from excessive forces [1–3]. Preservation of meniscal functions is essential, as the tissue’s degradation has been associated with the initiation and progression of osteoarthritis (OA) in the knee joint [4]. The investigation of meniscus properties and their relationship to composition and structure can help in further understanding of tissue pathophysiology, as well as in developing novel strategies for maintaining and restoring meniscus health.

Due to lack of blood flow, the meniscus has limited capability of self-repair [5–7]. At maturity, vascularity in the meniscus is restricted to the outer third of the tissue (red zone), leaving a majority of the tissue avascular (white zone) [5]. Accordingly, delivery of nutrients and larger proteins to and from the cells of the white zone relies on diffusion through the extracellular matrix (ECM) from the red zone [8, 9]. Previous studies have shown molecular diffusivity in the core of the meniscal tissue is anisotropic: faster in the direction parallel to the dominant circumferential fibers than in the perpendicular direction. It was also shown that the diffusivity is inversely related to the hydrodynamic radius of the diffusing molecule, following the Ogston model [6, 10–12]. Other studies on fibrocartilaginous tissues have shown solute transport across the ECM is dependent on tissue structure and composition [13–15]. It is well known that both structure and composition of the meniscus greatly vary across its regions. Specifically, the structure of its core region consists of bundles of collagen fibers oriented primarily in the circumferential direction [1, 16–20], whereas the structure of the surface layers (femoral and tibial) that wrap the core tissue are composed of randomly oriented collagen fibers, similar to the neighboring femoral and tibial hyaline cartilage [3, 20–22]. Additionally, the composition of the inner core region is approximately 75% water, 20% collagen fibers, and 5% non-collagenous substances including glycosaminoglycans (GAGs) [23], whereas the surface layers have significantly lower GAG and water contents [3, 22, 24, 25]. Differences in composition and structure have been shown to correlate with tissue mechanical properties [20]. In addition, previous studies have shown that diffusivity in cartilaginous tissues is inversely related to the molecular weight of the solute [6, 12, 14, 26–28].

Based on this experimental evidence, in this study we hypothesize that regional differences in diffusive transport coefficients will exist across meniscal core and surface layers. Additionally, we hypothesize that the diffusivity will decrease as the solute molecular weight increases for each region. Therefore, the objective of this study was to investigate the effect of meniscal region (core tissue, femoral, and tibial surface layers) and probe molecular weight on diffusivity.

Diffusivity measurements in porcine meniscal tissue were conducted via fluorescence recovery after photobleaching (FRAP), a technique used successfully in previous studies to investigate solute diffusivity in cartilaginous tissues [6, 12, 14, 15, 29–33]. The molecular solutes investigated in this study include fluorescein (F; MW 332 Da), due to its comparable size to nutrients, and fluorescence-labeled dextrans (3k Dex, 40k Dex, 150k Dex; MW 3k, 40k, 150k Da), representing medium and large molecules including proteins, growth factors, and potential molecular treatments for meniscal pathologies [11, 34, 35].

## Materials and methods

### Specimen preparation

**Fig 1:**
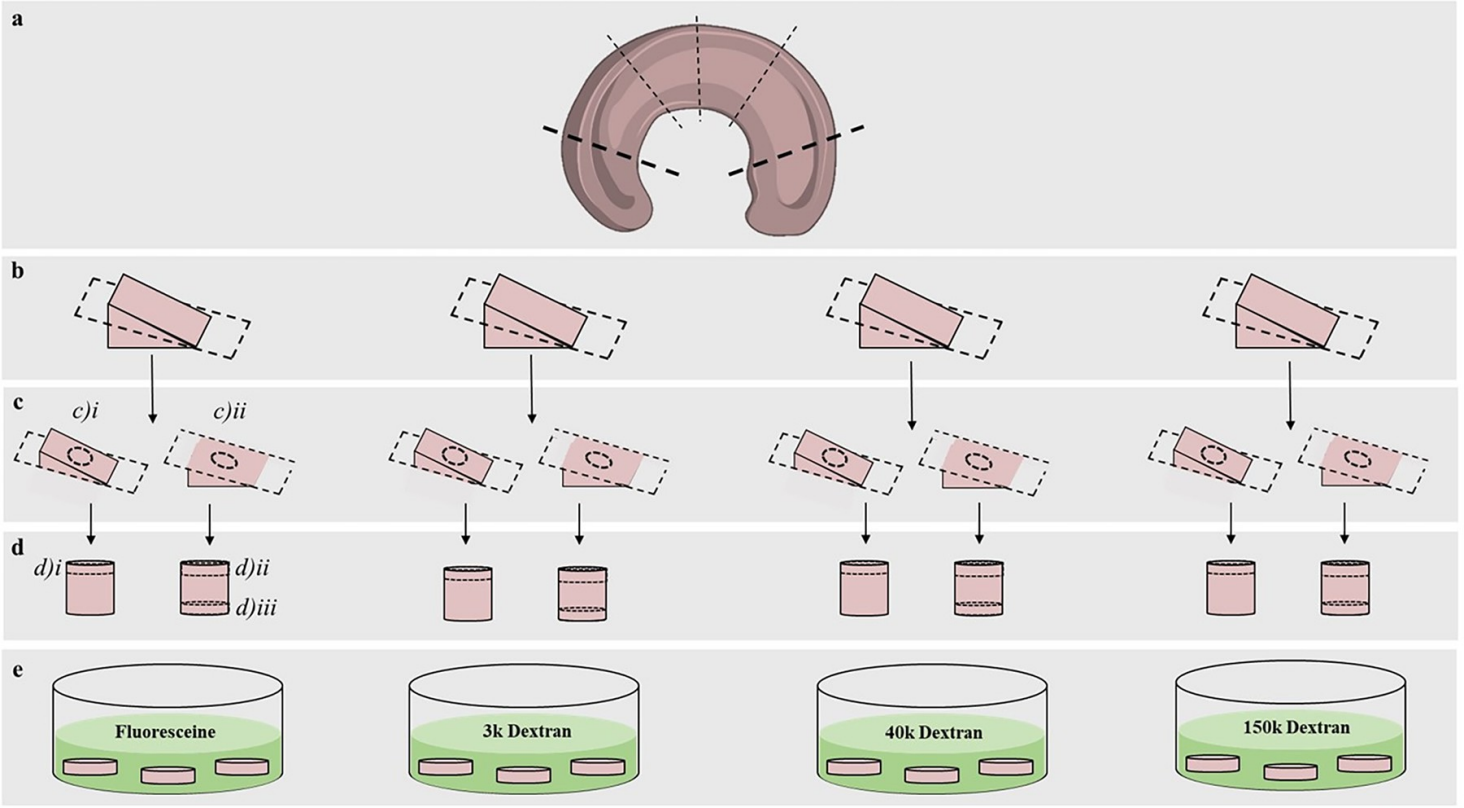
Schematic of specimen preparation. (a) The central region of a porcine meniscus was isolated and (b) cut into 4 equal parts. (c) Each part was cut parallel to the tibial surface layers to separate the (ci) femoral and (cii) tibial aspects. (d) Cylindrical samples were isolated from the center of tissue slabs and the (di) femoral surface, (dii) core, and (diii) tibial surface regions were cut to appropriate thickness. (e) Specimens were placed in solution containing the molecular probe of interest for equilibration.

**Fig 2:**
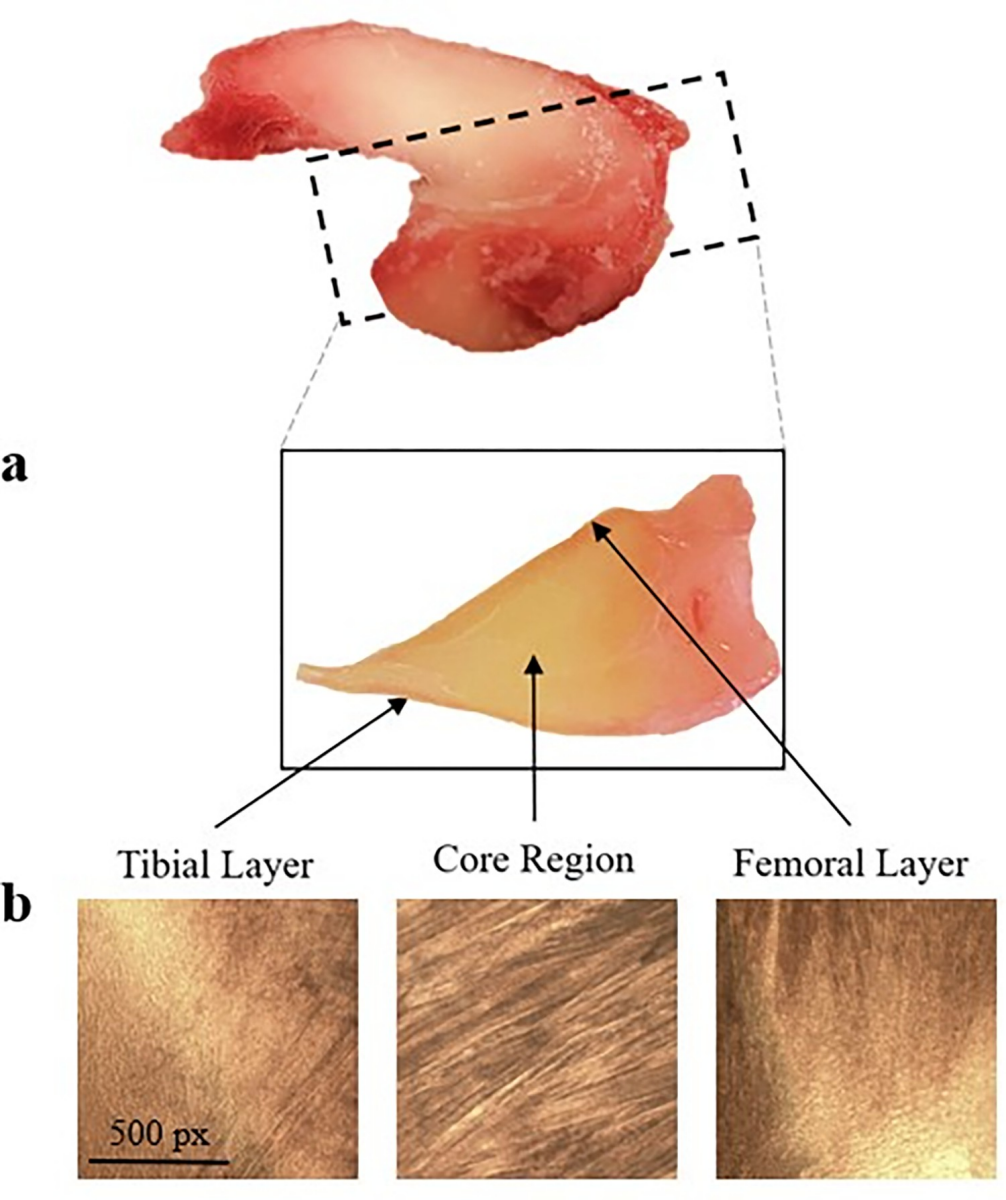
Visual differences between tissue regions. (a) A cut along the sagittal plane reveals the clear distinction between the core and its surface layers. (b) Visual inspection via light microscope of samples cut along the transverse direction confirmed their structural differences.

Porcine menisci (2+ years old) were obtained from a tissue bank (*Animal Technologies Inc*., *Tyler*, *TX*) and stored at -20°C until testing. The central region of 7 lateral menisci (n = 7) was isolated and each cut into 4 equal parts. Each part was cut in the middle, parallel to the tibial surface. With the surface layer of interest flat downwards, a 3mm corneal trephine was used to obtain tissue cylinders in the axial direction from the center of the tissue slab. Thus, specimens encompassed both the inner and outer regions of the meniscus, and the transition area joining these two. Meniscal cylinders were cut into 500μm thick discs using a custom 3D printed chamber and razor blade. A total of 12 samples were obtained from each meniscus, see Fig 1. Confirmation of region separation was done via light microscope (*VWR VistaVision*, *Radnor*, *PA*). Immediately after harvesting, each of the 4 equal parts of the meniscus and the samples pertaining to those sections, were randomly allocated and allowed equilibrate in a protease inhibited 1X phosphate buffer solution (PBS) solution (*Sigma-Aldrich Co*., *St*. *Louis*, *MO*) containing the molecular probe of interest, see Fig 2. Equilibration occurred overnight at 4°C. A total of four molecules were investigated. Specifically, diffusivities of 10μM F and FITC (fluorescein 5-isothiocyanate) labeled dextrans 3k Dex, 40k Dex, and 150k Dex were measured. A summary of molecular probes used in this study is reported in *Table 1*.

**Table 1:** Summary of molecular probes used in this study.

Molecular Probe	Abbreviation	Source	Molecular weight [Da]	Stokes’ radii, r^s^ (Å)	Diffusivity in water, D_o_ [μm^2^/s]
Fluorescein	F	Sigma-Aldrich	332	5[36]	425[37]
Dextran, Fluoresceine, 3000MW	3k Dex	Invitrogen	3,000	14[38]	155[39]
Dextran, Fluoresceine, 40,000MW	40k Dex	Invitrogen	40,000	45[38]	55[40]
Dextran, Fluoresceine, 150,000MW	150k Dex	Sigma-Aldrich	150,000	85[38]	25[39]

### Measurement of diffusivity

Solutes’ diffusion coefficients (*D*) within the tissue were measured via a custom FRAP technique [10]. It was assumed that diffusive fluorescence recovery would follow Fick’s law and occur in the focal plane (*x*,*y*) of the microscope objective. Also, the concentration of fluorescent probe was assumed to be proportional to the intensity of its fluorescent emission. Accordingly, the mass balance of the diffusing fluorescent probe follows:

$$\frac{\delta c}{\delta t}=\nabla ∙(D\nabla c)$$ (1)

where *c* is the molar concentration of the probe of interest and *D* is the diffusion coefficient. Eq (1) can be transformed and solved in the 2D Fourier space defined by the dimensionless frequencies (*u*,*v*) [41]:

$$\frac{C(u,v,t)}{C(u,v,0)}=exp[-4\pi^{2}(u^{2}+v^{2})Dt]$$ (2)

where *C(u*,*v*,*t)* is the 2D Fourier transform of *c(x*,*y*,*t)*. *D* is determined by curve-fitting the experimental data. This is represented by confocal microscope video images in Eq (2).

**Fig 3:**
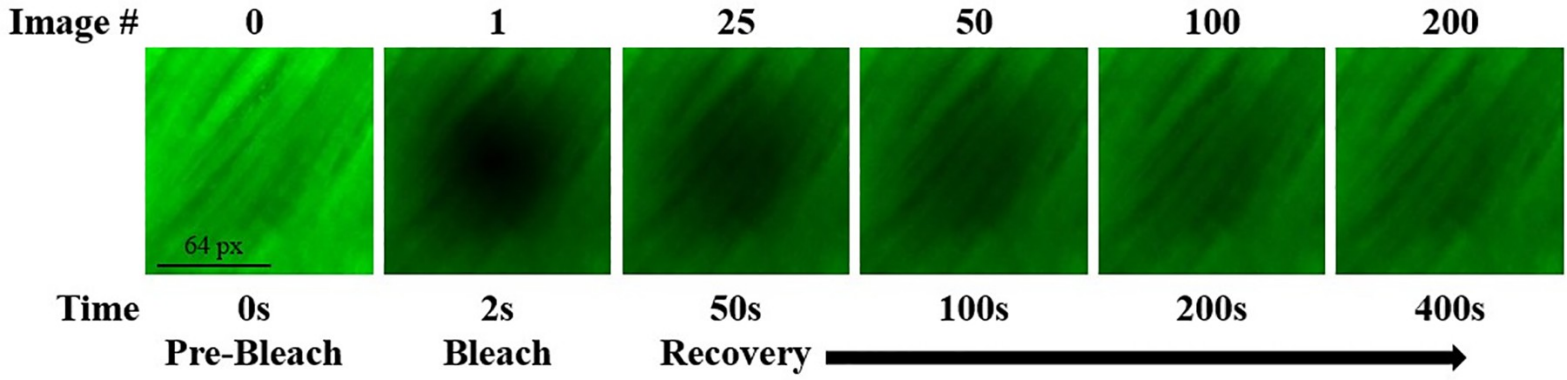
Representative images of FRAP process on a core sample. In image (0) the confocal microscope reveals the unbleached sample. Image (1) is the initial bleach spot followed by its recovery in images (25)-(200). Time values are reported below each image.

All experiments were conducted at room temperature (22°C) utilizing a confocal laser scanning microscope (*A1R-SI*, *Nikon*, *Japan*). Using an argon laser (wavelength 488nm) and a Plan Apo 20x/0.75 DIC N1 WD 1.0 objective (*Nikon*, *Japan*), each specimen was photobleached with an initial bleach spot with a 16-pixel diameter (~60μm). To mitigate errors due to out-of-plane diffusivity, a multi-layer bleaching protocol was implemented [12, 32, 42, 43]. Each test captured a time series of 200 video images sized at 128x128 pixels (460.7μm x 460.7μm) plus one image before the bleaching process. The time interval between two consecutive images was 2s, see Fig 3. Background fluorescence emission was minimized by subtracting the prebleach image from the postbleach image series. Three tests were performed and averaged on each specimen to account for structural variability within the tissue. Custom MATLAB®-based software was employed for image analysis.

### Statistical analysis

Minitab Statistical Software (*Minitab LLC*, *State College*, *PA*) was used to conduct statistical analyses. A two-way ANOVA with replication and factors being meniscal region (femoral surface, core, and tibial surface) and solute (fluorescein, 3k Dex, 40k Dex, and 150k Dex) was used to investigate possible significant interactions and main effects on the diffusion coefficient. Post hoc analysis via Tukey’s test was used to determine significant differences between individual groups. For all the analyses conducted, Grubbs tests were used to identify any outliers. Normality was verified via Anderson-Darling tests. For each test, a level of significance of 95% (α = 0.05) was used. Data are reported as mean ± standard deviation. Post hoc power analysis was conducted using G*Power (version 3.1.9.7) to find the statistical power the main effects considered.

## Results

**Fig 4:**
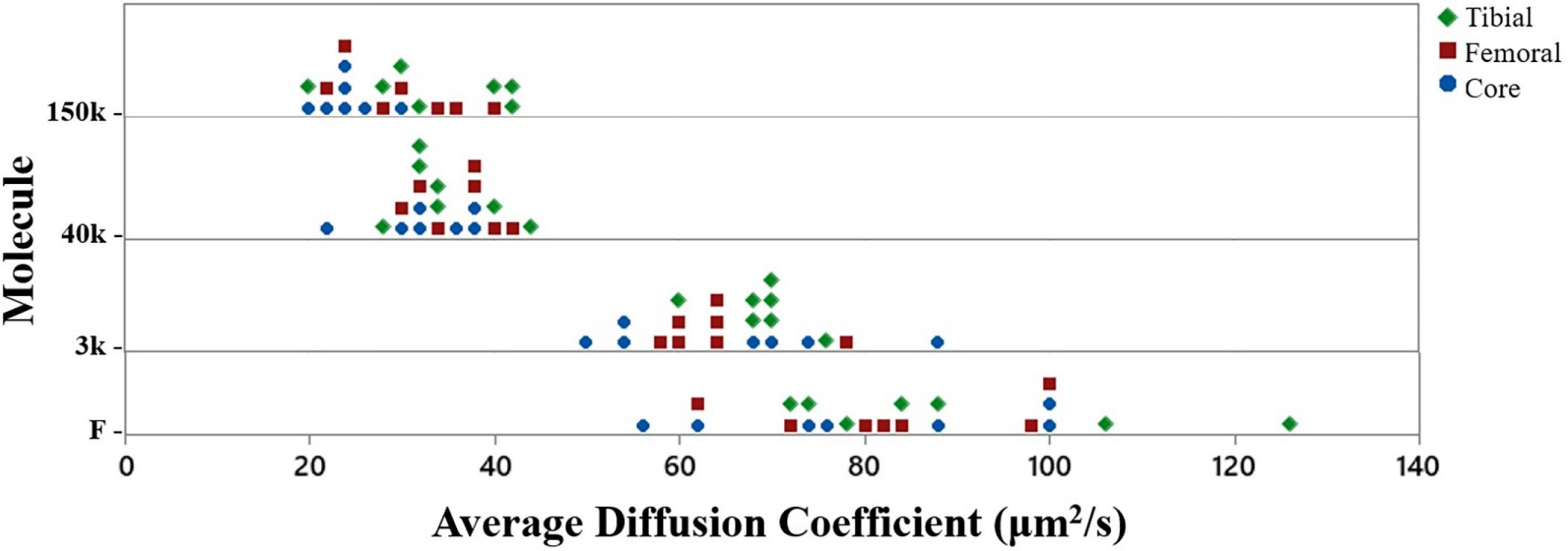
Summary diffusivity measurements. Diffusivity measurements of fluorescein (F), 3 kDa dextran (3k Dex), 40 kDa dextran (40k Dex), and 150 kDa (150k Dex) in porcine menisci (n = 7) subdivided by meniscal regions.

Average diffusivity measurements of solutes (F, 3k Dex, 40k Dex, and 150k Dex) for each region investigated (core tissue, femoral surface layer, and tibial surface layer) are shown in Fig 4. Average diffusivity ranged from 151.96 μm^2^/s for F to 10.71 μm^2^/s for 150k Dex. Two-way ANOVA revealed no interaction among main effects of molecular probe and meniscal region (p = 0.796). It was found that diffusivity decreased as molecular weight increased (p<0.001). When pooling data from all regions, Tukey post-hoc analysis showed significant differences between the diffusivities of all the probes (p<0.001), except between 40k Dex and 150k Dex (p = 0.193). Finally, no significant effect of meniscal region on diffusivity for F, 3k Dex, and 40k Dex was found. Conversely, region did significantly affect the diffusivity of 150k Dex: the diffusion coefficient in the tibial surface layer was significantly larger than in the core region (p = 0.001). There was no difference between the femoral surface layer and core region (p = 0.103) or between tibial surface layer and femoral surface layer (p = 0.272). Finally, post hoc power analysis indicated a power >95% in detecting significant effects among regions. Furthermore, an ANOVA for cross-validation purposes was conducted using a generalized linear model with a stepwise procedure. This model incorporated factors such as molecular probe, meniscal region, and meniscus, in addition to the covariate of cylindrical punch, taking into account all possible interactions. The results indicated that the meniscal region and the molecular probe both significantly affected the diffusion coefficient (p<0.05), aligning with prior findings. These results also revealed that meniscus and cylindrical punch had no significant effect on diffusion coefficient.

## Discussion

This study investigated diffusion of small to large sized solutes in three distinct regions of porcine meniscus (core, femoral, and tibial surface layers). Previous studies have recorded diffusivity of probes with a wide range of hydrodynamic radii in human and animal menisci [6, 10, 12]. In addition, molecular solubility has been studied across meniscal regions [26]. However, to the authors’ best knowledge, this is the first study reporting molecular and macromolecular diffusivities across different meniscal regions.

The average diffusion coefficients in the core region of F, 3k Dex, 40k Dex, and 150k Dex were 79.35±29.43μm^2^/s, 65.29±17.57μm^2^/s, 32.91±6.61μm^2^/s, and 24.64±7.32μm^2^/s, respectively. These findings are comparable to previous results of the same probes or similar sized molecules in porcine, human, and bovine menisci [6, 10–12]. With all three meniscal regions pooled together, larger molecules generally produced a lower diffusion coefficient. The diffusivity of fluorescein was the highest (83.69±25.33μm^2^/s), followed by 3k Dex (66.00±14.07μm^2^/s), 40k Dex (34.71±6.83μm^2^/s) and 150k Dex (29.20±8.37 μm^2^/s). Previous studies show a similar pattern: the magnitude of the diffusion coefficient is inversely related to the hydrodynamic radius of the diffusing molecule, particularly when normalized with respect to diffusivity in water (*D*_*o*_), the diffusion coefficients correlated to *r*^*s*^, follows a linear relationship [6, 10–12, 44]. These observed behaviors can be attributed to the steric interactions between the solute and collagen ECM: the diffusion of larger molecules within the tissue is limited by its tightly arranged ECM. This information can be leveraged for the design of molecular delivery treatments targeting various meniscal pathologies inside the tissue. By refining the size of therapeutic molecules, diffusivity throughout the inner meniscus could theoretically be optimized.

There was no effect of meniscal region on diffusivity of fluorescein, 3k Dex, and 40k Dex. However, region did significantly affect the solubility of 150k Dex: the diffusion coefficient of 150k Dex in the tibial surface layer was significantly larger than that in the core region (p = 0.001), see Fig 4. The ECM of the core region may hinder the diffusivity of such large molecules due to the size of its microtubes that run parallel to the fiber bundles [6, 12, 45]. In terms of developing molecular delivery treatments targeting meniscal pathologies of the avascular region which rely on diffusion through the ECM, this information indicates that the size of therapeutic molecules should also be considered in designing treatment interventions.

Limitations of this study should be noted. First, healthy porcine tissue was used in lieu of human samples. Although human samples have the highest clinical relevance, porcine tissue has shown to have strong similarities to that of human. Indeed, pigs have been successfully used as a surrogate model for the human meniscus [46–49]. In addition, all samples utilized were extracted from the central core region of lateral menisci only. Previous studies in porcine menisci have not found a difference between measured diffusivities in medial and lateral tissues [11, 50]. However, to obtain a more comprehensive understanding of the diffusive properties of the meniscus, future studies will examine potential additional regional differences (central vs horns, vascular (outer) vs avascular (inner)). Documented inconsistencies in the mechanical and transport properties of meniscus tissues across regions, related to regional variation in tissue composition, warrant the exploration of other regional variations [20, 51–53]. Finally, this study solely focused on unloaded tissue samples, while *in vivo* the tissue experiences diverse loading conditions including static and dynamic compression, shear, and tension. Prior research has demonstrated that the rate of solute transport in meniscus and articular cartilage is influenced by mechanical loading [12, 45, 54, 55]. To gain a deeper understanding of how such mechanical loading impacts solute transport through the tissue, further investigation is required. Nevertheless, the current study serves as a starting point, paving the way for more comprehensive exploration of solute diffusivity in the meniscus in future studies.

In conclusion, the effect of meniscal region (core tissue, femoral, and tibial surface layers) and molecular weight on the diffusivity of several molecules in the porcine lateral meniscus was investigated. For most molecules investigated, the diffusivity in the superficial layers was similar to that in the core of the tissue. This suggests that while diffusivity is not hindered by the meniscal superficial layers, size of the diffusing molecule must be considered for optimal efficiency of diffusion. Overall, this information adds new knowledge on the transport phenomena in the meniscus and can be leveraged to improve potential molecular delivery treatments for meniscal pathologies.

## Supporting information

S1 Dataset Comprehensive dataset. (XLSX)
